# Long-term efficacy of subthalamic nucleus deep brain stimulation in focal motor seizures and implications for candidate selection

**DOI:** 10.1016/j.neurot.2026.e00950

**Published:** 2026-06-24

**Authors:** Baoxin Xu, Xueyuan Wang, Xiaohua Zhang, Liang Qiao, Wei Shu, Duanyu Ni, Xiaoming Yan, Liankun Ren, Guoguang Zhao, Tao Yu

**Affiliations:** a Department of Neurosurgery, Xuanwu Hospital, Capital Medical University, Beijing, China; b Beijing Institute of Functional Neurosurgery, Beijing, China; c Department of Neurology, Xuanwu Hospital, Capital Medical University, Beijing, China

**Keywords:** Epilepsy, Focal motor seizures, Subthalamic nucleus, Deep brain stimulation

## Abstract

Focal motor seizures (FMS) are often unresectable because of motor risk. We aimed to evaluate the long-term effectiveness and safety of subthalamic nucleus deep brain stimulation (STN-DBS) in patients with FMS and to identify suitable candidate patients. We analyzed long-term outcomes in 22 patients with FMS treated with STN-DBS, classifying patients as responders (≥50% seizure reduction) or non-responders and relating outcomes to seizure-focus topography using focus-frequency maps and region-of-interest–based volumetrics. In a SEEG cohort of 13 patients with electrodes in the sensorimotor cortex and the STN, including 1 from the DBS cohort, we quantified changes in interictal spike (IIS) rates and broadband (0.5–90 Hz) power spectral density (PSD) across distinct sensorimotor subregions during 100-Hz STN stimulation. At final follow-up (mean 45 months), median seizure reduction was 55%, with 14/22 responders and six patients achieved >90% reduction, including one seizure-free. STN-DBS was well tolerated, with no surgical complications and one explant for infection. Responders’ seizure foci clustered in a medial sensorimotor strip comprising the paracentral lobule (PCL), supplementary motor area (SMA) and trunk representations of the precentral and postcentral gyri, and greater involvement of the PCL and trunk areas correlated with better outcome. SEEG analyses showed a global reduction in broadband power but regionally selective suppression of epileptiform activity, confined to the PCL and SMA. Together, STN-DBS appears to be a safe, effective option for FMS, and patients whose seizure foci involve the medial sensorimotor strip may be potential candidates.

## Introduction

Epilepsy affects approximately 1% of the population worldwide and constitutes a substantial public-health burden. Although many patients achieve satisfactory seizure control with antiseizure medications, roughly one third develop drug-resistant epilepsy (DRE) [1]. For a subset of these patients, resective surgery can provide durable remission [2,3]. However, focal motor seizures (FMS) arising from the central region, which are often frequent and stereotyped, pose a unique therapeutic dilemma because resection carries a high risk of irreversible functional deficits [[4], [5], [6], [7]]. Consequently, safe and effective non-resective therapies are urgently needed.

Over recent decades, neuromodulation has emerged as a promising therapeutic strategy for DRE. Established approaches include vagus nerve stimulation (VNS), responsive neurostimulation (RNS), and deep brain stimulation (DBS), and several DBS targets (for example, the anterior nucleus of the thalamus and hippocampus) have demonstrated benefit in selected cohorts [4,[8], [9], [10], [11]]. Nevertheless, dedicated evidence for the management of FMS remains limited: reports of VNS/RNS focused specifically on FMS are sparse, and commonly used thalamic targets do not consistently control motor-region seizures [8,12]. Accordingly, systematic, FMS-specific neuromodulation strategies that are explicitly tailored to the pathological networks remain to be established.

Neuromodulatory strategies guided by neural circuitry are receiving increasing attention [13,14]. From this perspective, FMS engage a cortico–basal ganglia–thalamocortical loop in which the subthalamic nucleus (STN) constitutes a key node whose stimulation can markedly modulate circuit function, a principle well established in the neuromodulation treatment of movement disorders [[15], [16], [17], [18], [19], [20], [21], [22]]. On this mechanistic basis, STN-DBS has been proposed as a candidate therapy for FMS. This hypothesis has been preliminarily supported by small case series reporting substantial seizure reductions following STN-DBS [[23], [24], [25], [26], [27]].

Complementing these clinical and preclinical observations, our prior intracranial electrophysiological studies in humans demonstrated that motor-cortex seizures recruit the ipsilateral STN, that single-pulse stimulation of STN contacts elicits clear, time-locked cortical evoked potentials, and that high-frequency STN stimulation can suppress cortical ictogenesis [28]. These findings supply mechanistic support for the hypothesis that STN-DBS could exert upstream modulation of motor-cortical epileptogenic circuits.

Concurrently, we conducted a cautious clinical evaluation of this hypothesis. Between 2013 and 2023, 22 patients at our center underwent STN-DBS. In this study, we analyzed their clinical characteristics and surgical outcomes both to validate our proposed network-based therapeutic hypothesis for focal motor seizures and to refine candidate-selection criteria by identifying the patient types most likely to benefit. Guided by findings from our clinical cohort and building on our previous report [28], we examined which sensorimotor subregions are most effectively modulated by high-frequency STN stimulation by quantifying changes in interictal spike rates and broadband power spectral density in a SEEG cohort. Overall, our aim was to evaluate the long-term clinical utility of STN-DBS for FMS, to identify practical preoperative biomarkers that could guide patient selection, and to begin to elucidate the network mechanisms that mediate therapeutic benefit.

## Materials and methods

### Patients

**Fig. 1:**
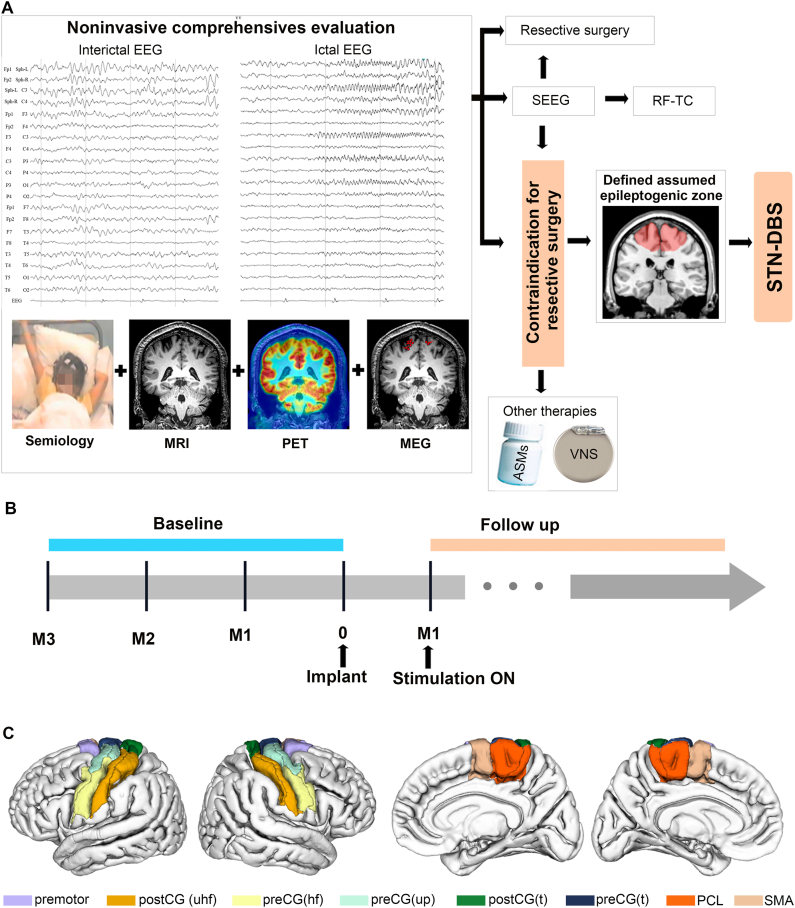
**Patient selection, DBS timeline, and ROI for focus-volume calculation.** A. Pipeline portrays the determining location of the seizure foci and patient selection. B. The time course of DBS surgery and stimulation programming. C. Regions of interest (ROIs) for focus-volume calculation. EEG: scalp video electroencephalography; MRI: magnetic resonance imaging; PET: positron emission tomography; MEG: magnetoencephalography; SEEG: stereo-encephalography; VNS: vagus nerve stimulation; RF-TC: radiofrequency thermocoagulation. M: month. postGC (uhf): upper limb, head and face representation of the postcentral gyrus; preGC (hf): head and face representation of the precentral gyrus; preGC (up): upper limb representation of the precentral gyrus; postGC (t): trunk representation of the postcentral gyrus; preGC (t): trunk representation of the precentral gyrus; PCL: paracentral lobule; SMA: supplementary motor region.

Between 2013 and 2023, a total of 2227 epilepsy-related neurosurgical procedures were performed at our center, from which we identified 22 consecutive patients who underwent STN-DBS. Patients were considered for STN-DBS after a comprehensive multidisciplinary presurgical evaluation (Fig. 1A) if they had drug-resistant FMS, a presumed epileptogenic zone involving motor-related eloquent cortex that made resective surgery unsuitable, and an epileptogenic region that was not sufficiently focal for SEEG-guided radiofrequency thermocoagulation (RF-TC). STN-DBS was also considered when disabling seizures persisted after prior RF-TC. Separately, we also reviewed 13 patients (including 1 patient from the DBS cohort) who underwent stereotactic implantation of depth electrodes in the STN and sensorimotor cortices. The patient selection criteria were consistent with those used in our previous study [28]. Written informed consent was obtained from all participants. The study protocol was approved by the Ethics Committee of Xuanwu Hospital, Capital Medical University (LYS[2024]297).

### Defining the seizure focus and ROI-based volumetrics

In this study of neuromodulation without resection, we adopt the term “seizure focus” as defined in our prior work [29]. The precise localization of the seizure foci was determined by the integrated, weighted seizure semiology, electrophysiology and neuroimaging for each patient. For each patient, an experienced epileptologist and a neurosurgeon (blinded to outcome) manually delineated the seizure focus on a standardized brain template in Montreal Neurological Institute (MNI) space using MRIcroGL (https://www.nitrc.org/projects/mricrogl). Discrepancies between raters were resolved by consensus. The group-level focus-frequency maps for responders and non-responders were generated by overlaying individual focus masks in the common MNI space.

Because epilepsy is predominantly a cortical disorder and to focus volumetric analysis on cortical tissue, the total focus volume for subsequent analyses was defined as the intersection between the hand-delineated seizure focus and a binary cortical mask derived from the Brainnetome (BN) atlas [30]. All focus masks and the BN atlas were resampled to a common MNI grid prior to intersection. The regions of interest (ROIs) in this study were defined by combining the corresponding BN parcels. All ROIs are presented in Fig. 1C, and their comprehensive definitions are provided in Supplementary Table S1. ROIs were then selected on the basis of group-level focus-frequency maps by identifying high-frequency loci. Since it is difficult to quantify the weighted contribution of multiple distinct foci within a single individual, seizure foci in each patient were merged into a single composite mask for the volumetric analyses. For each subject we computed the fraction of the total focus volume that fell within each selected ROI; for unilateral foci only ipsilateral ROIs were considered.

### DBS implantation

STN-DBS implantation was performed using frame-based stereotaxy (Radionics, USA) or a robotic stereotaxy arm (Sinovation Medical Technology, China). Quadripolar DBS leads (model 3389 diameter 1.27 mm, contact length 1.5 mm, intercontact spacing 0.5 mm, or 3387 diameter 1.27 mm, contact length 1.5 mm, intercontact spacing 1.5 mm, Medtronic Inc., Minnesota, USA) were targeted to the STN based on multimodal preoperative imaging and intraoperative physiology when available. Postoperative thin-slice CT/MRI were obtained for each patient and rigidly co-registered to the preoperative T1 MRI to confirm lead position.

### DBS programming and follow-up

All patients had prospective seizure counts documented for a 3-month baseline prior to implantation and stimulation was initiated one month after implantation (Fig. 1B). Conventional antiseizure medications were kept stable whenever clinically possible. Initial stimulation settings were monopolar configuration (implantable pulse generator as anode, selected electrode contact as cathode), pulse width 90 μs, frequency typically 130–145 Hz, and starting amplitude 1.0 V; amplitude and cycling parameters were then incrementally adjusted by the treating neurosurgeon according to patient tolerance and clinical response. The same programming principles were applied to unilateral and bilateral stimulation, with side-specific adjustments made when needed. Typical electrode impedances were approximately 1000 Ω (≈1 mA/V). The final stimulation settings are provided in Supplementary Table 1.

Follow-up visits were scheduled at 3, 6 and 12 months, and annually thereafter; each follow-up recorded the mean seizure frequency over the preceding 3 months. Each follow-up also record the stimulation parameters at that time. Side effects according to subjective reports during follow-up visits were documented. This study finally obtained follow-up records of all patients at 12 months, and before the study data were analyzed, a final follow-up was performed to assess long-term outcomes. If seizure reduction reaches 50% or greater than the baseline, the patient would be considered as a responder to STN-DBS, otherwise they would be classified as non-responders.

### Deep brain stimulation electrode localization

Lead-DBS software (http://www.lead-dbs.org) was used to localize the DBS electrodes [31,32]. In brief, the postoperative CT was linearly co-registered to the preoperative MRI using Advanced Normalization Tools (http://stnava.github.io/ANTs). Considering the possible brain shift owing to surgery, an additional subcortical refinement step - using the brain shift correction module in Lead-DBS – was employed to attain a precise subcortical alignment between pre- and postoperative images. Electrodes were reconstructed automatically using the PaCER algorithm [33].

### SEEG stimulation protocol

SEEG recordings used for the stimulation experiments were acquired while patients were awake and at rest. No clinical or electrographic seizures occurred during the 2-h window before or after stimulation. We applied a serial, incremental stimulation protocol on the STN consisting of frequencies from 10 to 130 Hz, a pulse width of 90 μs, and a stimulation intensity of 2 mA, delivered in a cycle of 60 s ON and 60 s OFF. The STN contact chosen for cathodal referential stimulation (with the adjacent contact serving as the anode) was the contact that exhibited the most prominent propagated ictal discharges. Concurrently, unstimulated SEEG contacts distributed across cortical regions were recorded continuously throughout the procedure.

### SEEG preprocessing and selection of contacts for analysis

SEEG data were processed off-line using EEGLAB and customized MATLAB codes (MathWorks, Natick, MA) unless otherwise indicated. Bipolar montage from the adjacent contacts of each SEEG electrode was routinely used to highlight local field potential (LFP) and reduce volume conduction. Moreover, LFPs were band-pass filtered (butterworth bandpass, zero-phase shift) in the range of 0.5–250 Hz unless otherwise indicated. The SEEG data were initially screened by visual inspection, and the epochs with significant artifacts were discarded for further analysis. For each subject, representative SEEG contacts situated within sensorimotor related cortical areas were chosen for analysis. Selection was restricted to contacts lying in gray matter, and adjacent contacts were avoided in order to reduce spatial redundancy and minimize volume conducted signal contamination. We then grouped the selected contacts into anatomically defined sensorimotor subregions using the BN atlas. The exact parcel composition of each subregion is provided in Supplementary Table 2.

### IIS detection and PSD estimation

To quantify pathological and physiological responses to STN stimulation, we measured IIS rate and PSD for each anatomically defined sensorimotor subregion. Because clinical STN-DBS is delivered at high-frequency stimulation (HFS), analyses were restricted to the 100-Hz stimulation epochs. Raw bipolar LFPs were band-pass filtered between 30 and 80 Hz (zero-phase) and rectified. Candidate IIS events were identified when the rectified signal amplitude exceeded the baseline mean by 4 standard deviations (channel-wise baseline computed from the 30-s pre-stimulation period). Candidate events with amplitude >10 baseline SD or inter-event intervals <40 ms were excluded to reduce false positives from transient artifacts. All automated detections were visually inspected on a per-recording basis to remove filter-induced artifacts and confirm accuracy. PSD values were averaged across the broadband range of 0.5–90 Hz, log-transformed as 10 × log10(PSD), and reported on a dB scale. For each stimulation trial we computed the IIS rate (events per second) and the relative change in mean broadband PSD between the 60-s stimulation epoch and the 30-s pre-stimulation baseline. We selected a 30s pre-stimulation baseline (rather than 60 s) to minimize contamination by delayed modulatory effects from any prior stimulation.

### Statistical analyses

Statistical analyses were performed with SPSS software, version 26.0 (IBM Corp., Armonk, NY, USA), and GraphPad Prism software, version 8.0 (GraphPad Software, San Diego, CA, USA). Continuous data were tested for normality using the Shapiro–Wilk test (p > 0.05 indicating approximate normality). Normally distributed data are reported as mean ± SD and non-normal data as median (IQR). Paired comparisons (stimulation vs baseline) used paired t-tests for normally distributed variables and Wilcoxon signed-rank tests for non-normal data. Between-group comparisons used two-sample t-tests or Mann–Whitney U tests, as appropriate. For categorical variables, frequencies are expressed as percentages. Depending on the data characteristics, either the chi-square test or Fisher's exact test was employed. Associations between proportional focus volumes and percent seizure reduction were assessed with Spearman rank correlations. Results are considered as exploratory, so no multiple comparison adjustment was performed. Statistical significance was defined as p < 0.05.

## Results

### STN-DBS patient characteristics

**Fig. 2:**
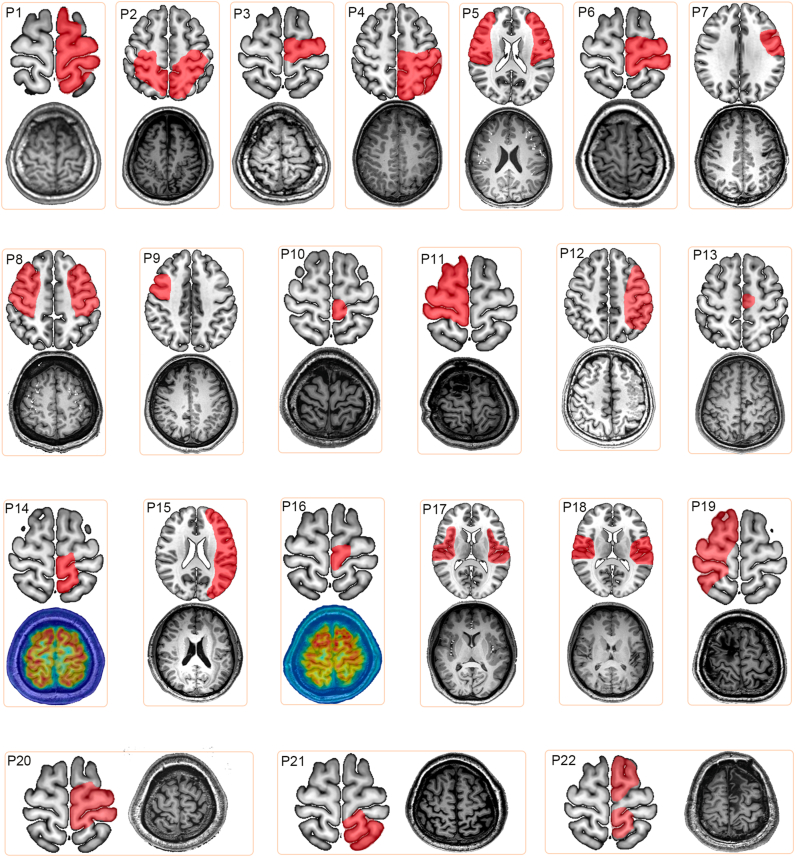
Seizure focus and primary localizing modality. Red overlays mark the seizure focus for patients 1–22, determined by a multidisciplinary presurgical evaluation. For each patient, the panel shows the modality that provided the primary focal evidence, PET for P14 and P16, MEG for P5 and P8, and MRI for the remainder. P: patient.

Ultimately, a total of 22 patients with DRE who underwent STN-DBS were included in this study (15 males and 7 females; mean age 21.3 ± 7.5 years; mean epilepsy duration 13.9 ± 6.4 years). Their median age at seizure onset was 7.0 years (IQR, 7.5), and the median baseline monthly seizure frequency was 37.0 (IQR, 121.1). MRI-visible structural abnormalities involving or adjacent to motor-related cortical regions were present in 17 of 22 patients. Ten patients had previously undergone epilepsy surgery or neuromodulation. Implantation was left-sided in 10 patients, right-sided in 2, and bilateral in 10. Regarding etiology, three patients had a history of encephalitis, one had hypoxic-ischemic encephalopathy, one had prior traumatic brain injury, 14 patients had cortical developmental malformations and three had epilepsy of unknown cause. Detailed demographic characteristics, clinical features, and non-invasive evaluation results are provided in Supplementary Table 3. We then compared baseline demographic and clinical variables between responder and non-responder groups. There were no significant between-group differences in age, gender, age at onset, disease duration, total focus volume, number of ASMs, or seizure semiology (see Table 1). For each patient, the seizure focus was manually traced onto a standardized MNI template representative localizing images (MRI, PET, or MEG) are shown in Fig. 2.

**Table 1:** Demographic and clinical characteristics of the patients.

Demographic and clinical characteristics	Responder(n = 14)	Non-responder(n = 8)	Test statistic (*T*, *Z or* χ^2^)	P Value
Sex			0.187	1.000
Male	10	5		
Female	4	3		
Age, years(Mean,SD)	22.6 (7.3)	19.1 (7.6)	−1.046	0.308
Age of epilepsy onset, years (Median, IQR)	7.0 (5.0)	3.0 (10.0)	−1.342	0.179
Duration of epilepsy, years (Mean, SD)	13.3 (5.3)	14.1 (7.0)	−0.312	0.758
Total focus volume, cm^3^ (Mean, SD)	59.5 (55.5)	120.8 (85.5)	2.046	0.054
Seizure frequency, monthly, log, (Mean, SD)	0.62 (0.67)	0.80 (0.67)	−1.039	0.303
ASMs, (median, IQR)	2.0 (1.0)	2.0 (1.0)	−1.057	0.291
Seizure classification				
Tonic	5 (35.7%)	5 (62.5%)	1.473	0.613
Tonic-clonic	10 (71.4%)	6 (75.0%)	0.033	1.000
Myoclonic	2 (14.3%)	1 (12.5%)	0.014	1.000
Epileptic spasm	2 (9.1%)	0 (0.0%)	1.257	0.515
Postoperative complications				
Headache		1		
Infection	1			
Paresthesia		1		
Psychiatric symptoms	1			

IQR: interquartile range; SD: standard deviation; y: years; ASMs: antiseizure medications; Categorical variables were compared using Fisher's exact test or chi-square test (reporting χ^2^ statistic). Continuous demographic variables with normal/non-normal distributions are presented as mean (SD)/median (IQR), with corresponding T-statistics or Z-statistics reported accordingly.

### STN-DBS follow up outcome

**Fig. 3:**
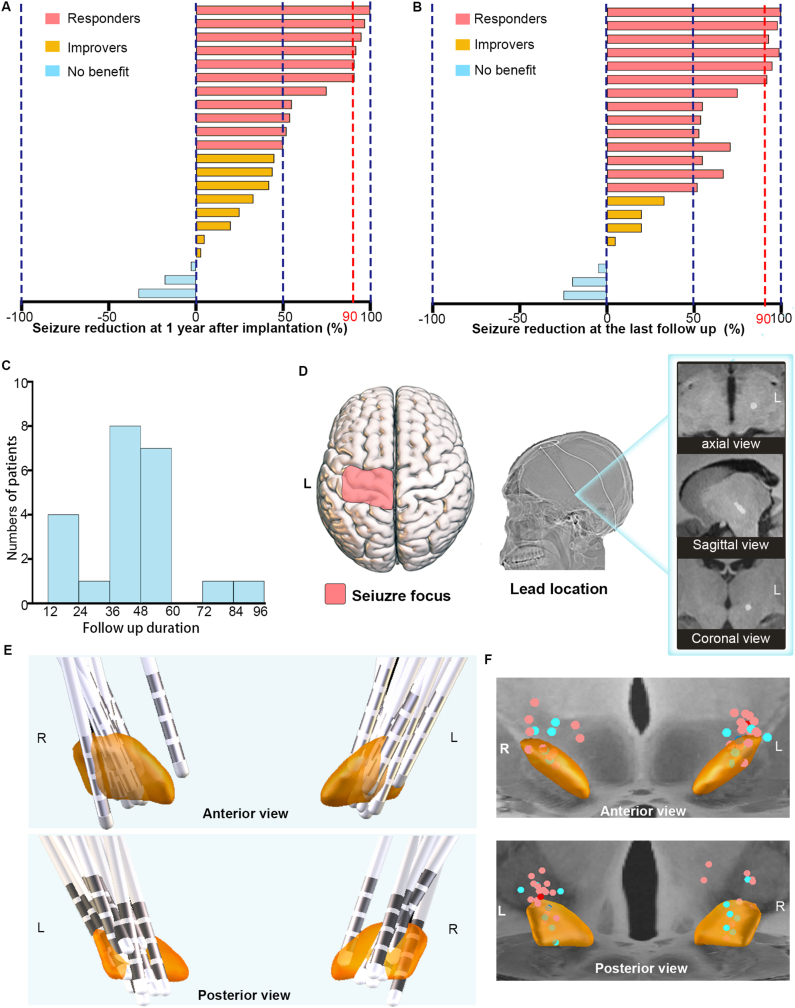
**Follow-up outcomes and electrode location. A.** Seizure reduction at 1 year after surgery. **B.** Seizure reduction at final follow-up. **C.** Duration of follow-up. **D.** Seizure focus and lead location in a representative patient**. E.** The posterior and anterior view of 3D grouped rendering of the normalized reconstructed lead trajectories and DBS-electrode contacts over the STN (show in orange). **F.** Light blue and light red colors represent non-responders vs. responders, respectively and dark blue vs. dark red circles depict the two patients with the least and most clinical benefit. Improvers: seizure reduction >0% and <50%. L: left; R: right.

All patients completed at least one year of follow-up. At one year follow-up, the median (IQR) seizure reduction was 48% (IQR 21–87%), and 11 of 22 patients (50%) were responders. Six patients(27.3%)achieved >90% reduction and one was seizure-free. Among non-responders, eight experienced some reduction in seizure frequency compared with baseline, whereas three derived no benefit from STN-DBS (Fig. 3A). No intraoperative or immediate postoperative surgical complications were observed. Two patients experienced stimulation-related adverse effects: one had headache with contralateral upper-limb dyskinesia, and one developed anxiety symptoms (see Table 1). These stimulation-related effects largely resolved with ongoing parameter adjustment. At the final follow-up (mean 45 months), the median (IQR) seizure reduction among all 22 patients was 55% (20–88%), with 14 (63.6%) classified as responders (Fig. 3B). The distribution of follow-up durations is shown in Fig. 3C. Six patients maintained >90% reduction and one remained seizure-free. Three patients who were non-responders at one year (with reductions of 42%, 44% and 45%) converted to responders by final follow-up (52%, 67% and 55%, respectively). Conversely, eight patients were consistent non-responders. Among these, four had some reduction that remained below the responder threshold, and four showed no benefit.

By the last assessment, 17 patients were followed-up for at least 3 years. After the first year, 4 patients turned off the device because of insufficient power or poor outcome. One patient developed a device-related infection 44 months after surgery and underwent explantation of the pulse generator and leads. During follow-up, 5 patients added a new ASM, 2 switched from one ASM to another, and 5 increased the dose of an existing ASM. Final stimulation parameters did not differ significantly between responders and non-responders (Supplementary Material). The Fig. 3D illustrates the seizure focus and the left-sided STN stimulation site in a representative patient who achieved seizure freedom at one year and remained seizure-free at the final follow-up (32 months). Normalized electrode locations and active contacts for all subjects are shown in Fig. 3E and F.

### Predictors of response to STN-DBS

**Fig. 4:**
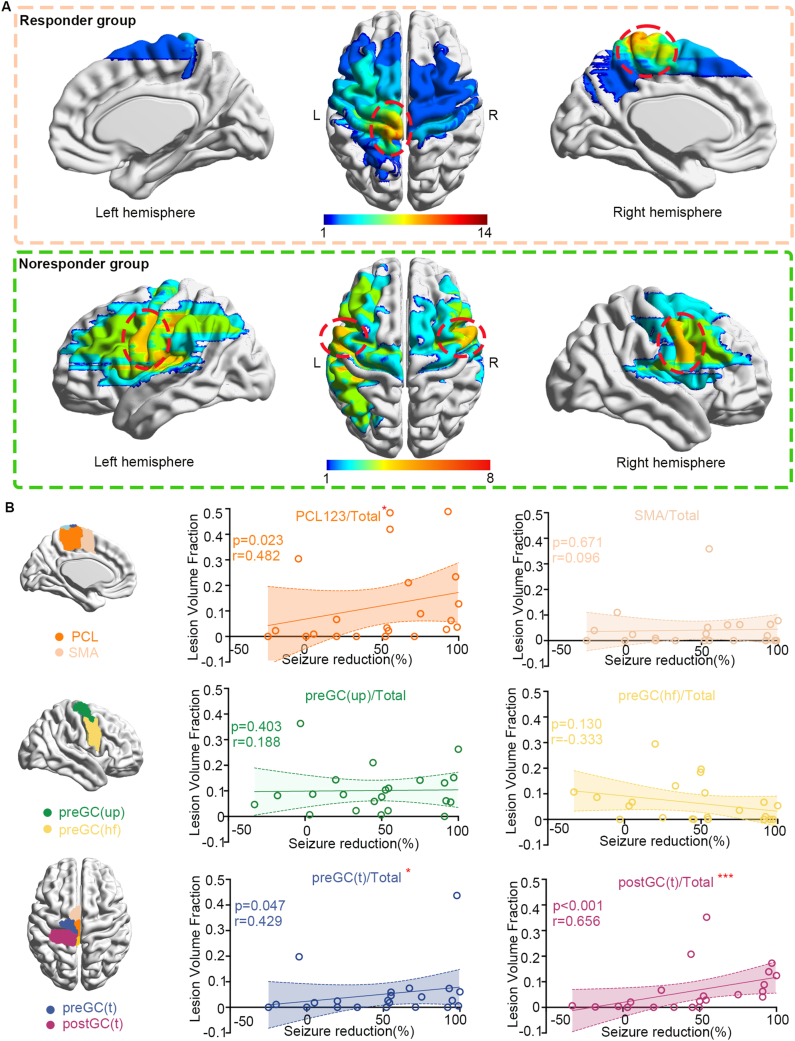
**Focus-frequency maps and associations between seizure-focus spatial distribution and outcome. A.** Focus-frequency maps for responder and non-responder groups projected onto a 3D template; the color bar indicates the number of patients whose focus mask includes the voxel. **B.** Scatter plots showing correlations between the proportion of total focus volume within each predefined sensorimotor subregion and percent seizure reduction at final follow-up. L: left; R: right; ∗:P < 0.05, ∗∗∗:P < 0.001.

Focus-frequency maps for responders and non-responders at final follow up were overlaid and projected onto a 3D template for visualization (Fig. 4A). The maps revealed that responders' high-frequency foci clustered within the medial sensorimotor strip, including the paracentral lobule (PCL), the trunk representations of the precentral (preCG, t) and postcentral (postCG, t) gyri, and the supplementary motor area (SMA). Because 64.3% (9/14) of responders had left-sided foci, these clusters were predominantly left-lateralized. In contrast, non-responders’ high-frequency regions were concentrated in the orofacial (preCG, hf) and upper-limb (preCG, up) representations of the precentral gyrus.

To test whether seizure-focus topography predicted outcome, we computed, for each predefined high-frequency subregion, the fraction of a patient's total focus volume that lay within that subregion and correlated these proportions with percent seizure reduction at final follow-up (Fig. 4B). Greater focus proportion in the PCL (r = 0.482, P = 0.023), preCG (t) (r = 0.429, P = 0.047) and the postCG_t (r = 0.656, P < 0.001) was associated with better outcome. Proportions for the SMA, and preCG (up) regions showed trends toward association with outcome but did not reach statistical significance (P > 0.05). Notably, the preCG (hf) proportion trended negatively with outcome, although this did not attain significance. The correlation trends were broadly consistent at one year post-surgery, except that the precentral trunk proportion did not reach statistical significance (see Supplementary Fig. 1).

### STN high-frequency stimulation modulates sensorimotor subregions

**Fig. 5:**
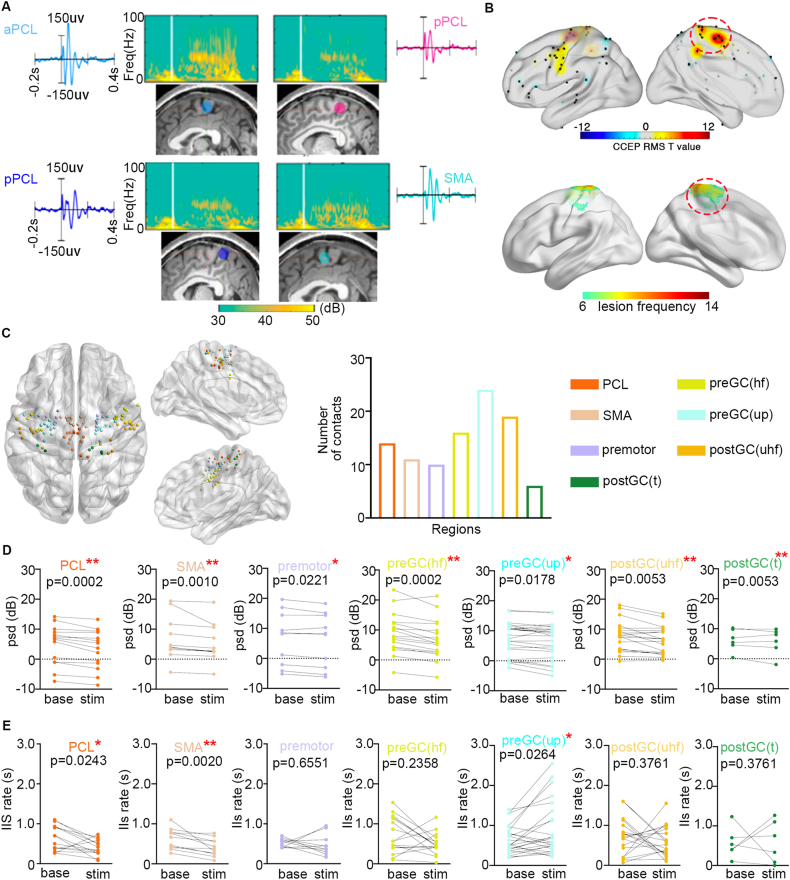
STN stimulation modulates sensorimotor subregions. A. The paired display of the spectrogram of ictal discharges and averaged STN cortical evoked potentials on representative contacts (adapted from Ren et al. [28] with modifications, adapted panels used with permission). **B.** Spatial map of RMS t value of STN-evoked cortical potentials (adapted from Ren et al. [28]) and focus-frequency of responder group on Montreal Neurological Institute (MNI) standard template. C. The locations and number of electrode contacts in each subregion. **D.** Comparison of broadband (0.5–90 Hz) mean PSD between stimulation and baseline across subregions. E: Comparison of interictal spike rates between stimulation and baseline across subregions. ∗: P < 0.05; ∗∗: P < 0.01.

We previously validated motor cortico-subthalamic effective connectivity by recording cortico-cortical evoked potentials (CCEPs) elicited by single-pulse stimulation of the STN [28]. In representative patients, single-pulse stimulation of SEEG electrodes implanted in the STN produced clear, time-locked cortical evoked potentials with the largest amplitudes located in SMA and PCL (Fig. 5A). At the group level, root mean square (RMS) t values of CCEPs in this region were significantly greater than baseline, and this activation overlapped the high-frequency focus regions identified in responders (Fig. 5B).

Extending our prior observation that STN stimulation differentially modulates motor versus non-motor cortex, we examined the effects of 100-Hz STN stimulation across anatomically defined sensorimotor subregions (PCL, SMA, premotor cortex, preCG (hf), preCG (up), postCG (uhf), postCG (t), see Fig. 5C). For each subregion we quantified changes in IIS rate and in broadband PSD during stimulation relative to a prestimulus baseline. STN-HFS produced a robust reduction in broadband PSD across all sensorimotor subregions, indicating a widespread physiological suppression of cortical power (Fig. 5D). By contrast, suppression of pathological activity was regionally selective: significant reductions in IIS rate were confined to medial central-sulcus regions (PCL and SMA) (Fig. 5E). The precentral upper-limb representation paradoxically showed an increase in IIS during stimulation, although sensitivity analysis suggested that this finding may have been influenced by outlier contacts (Supplementary Material). These findings suggest that although STN-HFS produces broad physiological modulation across the sensorimotor cortex, its suppression of pathological epileptiform activity is regionally specific.

## Discussion

### Clinical validation of STN-DBS for FMS

FMS arising from motor-cortex networks cause considerable disability, and resective surgery often risks irreversible functional loss when epileptogenic tissue involves the primary motor cortex, PCL or SMA [34]. Motivated by prior network studies and our earlier intracranial electrophysiological findings, we hypothesized that STN-DBS may provide an effective, non-resective treatment option for these patients. This study reports the clinical validation of that hypothesis. In this single-center series of 22 consecutive patients with drug-resistant FMS, STN-DBS produced durable seizure reduction with an acceptable safety profile: at final follow-up the median seizure reduction was 55%, six patients achieved >90% reduction (one became seizure-free), and no intra- or immediate postoperative surgical complications were observed. Stimulation-related adverse events were transient and improved with programming. These results align with prior small case series and support STN-DBS as a reversible alternative to resection for selected patients with motor-region epilepsy [24,26,27,[35], [36], [37], [38]].

Unlike RNS and ANT-DBS, STN-DBS in our cohort exhibited a three-tier pattern of response. A minority of patients experienced an early, dramatic benefit of >90% seizure reduction within the first postoperative year, generally sustain excellent long-term outcomes and often require less aggressive reprogramming. A larger subgroup achieved partial improvement, commonly >40% reduction at one year, frequently gain further improvements with careful, prolonged parameter optimization, although these gains rarely reach the magnitude seen in the early dramatic responders. Given high baseline seizure burden, even partial improvement yielded meaningful functional and quality-of-life gains. By contrast, patients with minimal early benefit rarely converted to responders. Given that focal motor seizures were often very frequent, even 50% reduction yields major functional gains. These results emphasize the critical need to discover robust prognostic biomarkers and to refine pre-implantation candidate-selection criteria for STN-DBS.

### Predictors of response to STN-DBS

The identification of reliable prognostic predictors has long been a key issue in the field of neuromodulation for epilepsy [14]. Previous studies have reported associations between preoperative measures and response to RNS, ANT-DBS and VNS [29,39,40]. However, predictors specific to STN-DBS for epilepsy remain very limited, leaving epilepsy surgeons without evidence to guide patient selection. In this study, we compared the seizure focus distributions between two groups with different outcomes at the final follow-up and found that the responders' foci were clustered on the medial sensorimotor strip. Further correlation analyses showed that the proportion of total focus volume within the PCL and postCG (t) was significantly associated with postoperative outcome. To our knowledge, systematic data on pre-implantation predictors of response to STN-DBS for epilepsy are scarce, and our results suggest that such factors may have potential predictive and clinical value.

Notably, the high-frequency focus region in the responder group spatially overlapped with the cortical sites showing the high RMS-t values in cortico-cortical evoked potentials (CCEPs) elicited by STN single-pulse stimulation. This spatial concordance provides preliminary support for STN-DBS candidate selection: when an epileptogenic focus lies within cortex that is strongly and directly coupled to the STN, high-frequency STN stimulation is more likely to engage and modulate the epileptogenic circuit.

### Regional specificity of STN-HFS effects on the sensorimotor cortex

Our SEEG cohort provides a mechanistic context for our clinical findings. Understanding the mechanisms of neuromodulation in epilepsy is crucial for patient selection and the development of precision therapeutic strategies. While DBS, VNS and RNS have all demonstrated clinical benefit in selected cohorts, the mechanistic basis for their effects remains incompletely defined. In particular, mechanistic data on STN-targeted stimulation for epilepsy are scarce compared with other DBS targets (ANT, CMT, hippocampus) [40,41]. Here, we subdivided the sensorimotor cortex into anatomically defined subregions and tested the effects of high-frequency STN stimulation on each subdivision.

Our electrophysiological results revealed a clear dissociation. High-frequency STN stimulation produced widespread suppression of broadband (0.5–90 Hz) power across most sensorimotor subregions. By contrast, the effect on pathological discharges (IIS) was region-specific. Significant IIS reductions were observed in medial central regions (the PCL and the SMA), whereas no significant IIS suppression was detected in convexity sensorimotor areas. This dissociation indicates that, although broad physiological power reductions or disruption of rhythmic activity may provide a permissive substrate for suppressing pathological discharges, they are not sufficient to ensure focal suppression, which may also depend on local circuit architecture and plasticity, and on the strength and topology of STN-cortex effective connectivity.

Recent studies on the spatial localization of DBS treatment effects provide interesting parallels for our findings. On one hand, Horn et al. established structural and functional connectivity models in a cohort of Parkinson's disease patients undergoing STN-DBS, revealing that the connectivity profile associated with clinical improvement included strong structural connections with the supplementary motor area and other frontal motor-related regions, as well as functional anti-correlations with the primary motor cortex [31]. Meanwhile, a recent study specifically focused on DBS for tremor symptoms integrated findings from lesion network mapping, fMRI, atrophy analysis, and DBS network mapping to generate a multi-modal, data-driven integrated tremor map. Intriguingly, within this integrated network, the region showing the strongest positive correlation with tremor relief was located in the medial portion of the sensorimotor strip [42]. These observations prompt the hypothesis that the STN, together with medial sensorimotor strip, may form pivotal network nodes for involuntary movement phenomena, a possibility that warrants dedicated multi-disorder network mapping studies.

This study has several limitations. First, the seizure-focus masks were manually drawn based on the clinical hypothesis, and the etiological heterogeneity of the cohort may complicate the interpretation of network effects and limit the generalizability of our findings. In addition, unlike Parkinson's disease and tremor, the therapeutic response to STN-DBS in epilepsy is likely influenced by several factors, including seizure focus location, variable placements of DBS leads and individual epilepsy networks. Consequently, it remains challenging to determine the optimal stimulation site within the STN. Antiseizure medication adjustments during follow-up may represent a potential confounder when interpreting the long-term efficacy of STN-DBS. Because intracranial recordings are invasive and electrode coverage varied across patients, electrophysiological recordings were not available from every sensorimotor subregion in every subject. Furthermore, the absence of multiple-comparison correction in these exploratory analyses may have increased the risk of Type I error. Finally, findings related to candidate selection and predictive biomarkers should be considered hypothesis-generating and require validation in larger prospective studies.

In summary, our study suggests that STN-DBS can provide safe and durable seizure reduction in patients with FMS. The ability of high-frequency STN stimulation to suppress epileptiform activity appears region-dependent, and patients whose seizure foci involve the medial sensorimotor strip may be potential candidates for STN-DBS. These findings require validation in larger prospective studies.

## Author contributions

Baoxin Xu: Data curation, Formal analysis, Investigation, Methodology, Software, Visualization, Writing – original draft, Writing – review & editing. Xueyuan Wang: Data curation, Formal analysis, Methodology, Software, Validation, Writing – original draft, Writing – review & editing. Xiaohua Zhang: Data curation, Supervision, Writing – original draft, Writing – review & editing. Liang Qiao: Methodology, Supervision, Writing – original draft, Writing – review & editing. Wei Shu: Data curation, Supervision, Writing – original draft, Writing – review & editing. Duanyu Ni: Data curation, Supervision, Writing – original draft, Writing – review & editing. Xiaoming Yan: Data curation, Investigation, Writing – original draft, Writing – review & editing. Liankun Ren: Conceptualization, Supervision, Writing – original draft, Writing – review & editing. Guoguang Zhao: Conceptualization, Supervision, Writing – original draft, Writing – review & editing. Tao Yu: Conceptualization, Funding acquisition, Project administration, Resources, Supervision, Writing – original draft, Writing – review & editing.

## Data availability

The data that support the findings of this study are available from the corresponding author upon reasonable request.

## Funding

This work was supported by the Brain Science and Brain-like Intelligence Technology - National Science and Technology Major Project (grant numbers 2021ZD0201605), the Beijing Municipal Natural Science Foundation (grant numbers L256008), the Beijing Municipal Health Commission Research Ward Excellence Clinical Research Program (grant numbers BRWEP2024W022010202).

## Declaration of competing interest

The authors declare the following financial interests/personal relationships which may be considered as potential competing interests: Tao Yu reports financial support was provided by Ministry of Science and Technology of the People's Republic of China. Tao Yu reports financial support was provided by Beijing Municipal Natural Science Foundation. Tao Yu reports financial support was provided by Beijing Municipal Health Commission. If there are other authors, they declare that they have no known competing financial interests or personal relationships that could have appeared to influence the work reported in this paper.
