# Supplementary material for: Long-term efficacy of subthalamic nucleus deep brain stimulation in focal motor seizures and implications for candidate selection
Source: Neurotherapeutics. 2026 Jun 24;23(4):e00950. doi: 10.1016/j.neurot.2026.e00950 (PMC13316762; doi:10.1016/j.neurot.2026.e00950)
Supplement: Multimedia component 1 [file mmc1.docx]

**Comparison of final stimulation parameters between responders and non-responders**

We compared the final stimulation parameters between responders and non-responders at the last follow-up. Pulse width was 90 μs in all patients and was therefore not statistically compared. Stimulation frequency was not significantly different between responders (median, 130 Hz; IQR, 0 Hz) and non-responders (median, 130 Hz; IQR, 0 Hz) (P=0.171). For bilateral stimulation, the same stimulation frequency was used on both sides in all patients. For amplitude comparison, the mean of the left and right amplitudes was used for bilateral stimulation when the two sides differed. One patient programmed in current-control mode was excluded from the voltage-amplitude comparison because current could not be reliably converted to voltage without contact-specific impedance data. Stimulation amplitude was not significantly different between responders (median, 1.20 V; IQR, 0.75 V) and non-responders (median, 1.30 V; IQR, 0.50 V) (P=0.466). A sensitivity analysis using the higher amplitude in bilateral cases yielded the same conclusion (P=0.661). The proportion of patients receiving bilateral stimulation also did not differ significantly between groups (8/14 vs. 2/8; P=0.204). Overall, no significant differences in final stimulation parameters were observed between responders and non-responders.

**Supplementary sensitivity analysis**

Because the precentral upper-limb region was the only sensorimotor subregion showing a paradoxical increase in IIS activity during stimulation, we performed a targeted sensitivity analysis for this region to assess whether the finding was driven by extreme contact-level values. IIS change was calculated as stimulation minus baseline. Contact-level outliers were identified using the 1.5 × interquartile range rule based on ΔIIS values. Two contacts were identified as outliers and excluded from the sensitivity analysis. After outlier exclusion, IIS activity remained numerically higher during stimulation than at baseline, but the difference no longer reached statistical significance (P=0.0838), suggesting that the apparent increase may have been influenced by outlier contacts.

**Supplementary Table 1.** Patient-level STN-DBS contacts, stimulation parameters, follow-up, seizure reduction, and complications

| P | Active contact (L/R) | Stimulation parameters  (Pulse width (μsec)/ Frequency (Hz)/ Amplitude(V) or ****Electric current (mA)/**** ****Pattern)**** | FU duration (months) | Seizure reduction (1year/last, %) | ****Complications**** |
| --- | --- | --- | --- | --- | --- |
|  | 10-C+ (L） | 90/160/0.5mA/ **continuous stimulation** | 85 | 91/95 | none |
|  | 2-C+/10-C+ | 90/130/2V/ ON: OFF 1:5min | 77 | 92/99 | none |
|  | 9-C+ (L） | 90/ 130/ 2.6V /ON: OFF 1:3min | 55 | -3/-5 | none |
|  | 9-C+ (L) | 90/ 130/ 0.9V /ON: OFF 1:5min | 40 | 25/20 | none |
|  | 1-0+/9-8+ | 90/ 130/ 1.2V/ON: OFF 1:5min | 13 | -18/-20 | none |
|  | 9-C+ (L) | 90/ 130/ 0.7V/ON/OFF=1:5min | 57 | 44/67 | none |
|  | 1-C+/10-C+ | 90/ 130/ R1V，L0.8V/ON:OFF 1:5min | 57 | 50/71 | none |
|  | 11-C+/3-C+ | 90/ 130/ 1.6V/ON: OFF 1:5min | 44 | 52/53 | **Infection** |
|  | 1-0+ (R) | 90/ 60/ 1.2V/ **continuous stimulation** | 55 | 20/20 | none |
|  | 2-3+/8-9+ | 90/ 130/ 2.1V/ON:OFF 1:5min | 52 | 55/55 | none |
|  | 3-2+/11-10+ | 90/ 130/ L0.8V R2V/ON:OFF  1:5min | 51 | 75/75 | none |
|  | 10-C+ (L) | 90/ 130/ 1.4V/ON:OFF  1:5min | 49 | -33/-25 | **Right lower limb paresthesia.** |
|  | 9-C+ (L) | 90/ 130/ 1.4V ON:OFF 1:5min | 46 | 45/55 | none |
|  | 10-C+ (L) | 90/ 130/ 1.2V ON:OFF 1:5min | 44 | 95/93 | none |
|  | 2-C+  (left) | 90/ 130/ 1.7V/ON:OFF 1:5min | 42 | 3/0 | none |
|  | 2-C+（left） | 90/ 130/ 0.5V/ON:OFF 1:5min | 41 | 97/98 | none |
|  | 2-C+  / 10-C+ | 90/ 130/1V(L),0.7V(R)/ 1:5min | 40 | 33/33 | none |
|  | 2-C+  / 10-C+ | 90/ 130/1V(L),0.7V(R)/ 1:5min | 38 | 42/52 | **Headache and psychiatric symptoms** |
|  | 1-C+（right） | 90/130/1.6V/1:5min | 21 | 5/5 | none |
|  | 1-C+（left） | 90/130/1.6V/1:5min | 32 | 100/100 | none |
|  | 1-C+  / 9-C+ | 90/130/1V(L),0.7V(R)/1:5min | 21 | 91/92 | none |
|  | 1-C+  / 10-C+ | 90/130/1V/1:5min | 21 | 54/54 | none |

P:patient; L:left; R:right; FU:follow up

**Supplementary Table 2.** Brainnetome parcel composition of regions of interest (ROIs)

| **ROI** | **Left and Right Hemisphere** | **Label ID.L** | **Label ID.R** | **Anatomical and modified Cyto-architectonic descriptions** | **lh.MNI(X,Y,Z)** | **rh.MNI(X,Y,Z)** |
| --- | --- | --- | --- | --- | --- | --- |
| precentral gyrus (head and face) | PrG_L(R)_6_1 | 53 | 54 | *A4hf, area 4(head and face region)* | -49, -8, 39 | 55, -2, 33 |
|  | PrG_L(R)_6_6 | 63 | 64 | *A6cvl, caudal ventrolateral area 6* | -49, 5, 30 | 51, 7, 30 |
| precentral gyrus (upper limb) | PrG_L(R)_6_3 | 57 | 58 | *A4ul, area 4(upper limb region)* | -26, -25, 63 | 34, -19, 59 |
|  | PrG_L(R)_6_2 | 55 | 56 | *A6cdl, caudal dorsolateral area 6* | -32, -9, 58 | 33, -7, 57 |
| precentral gyrus (trunk) | PrG_L(R)_6_4 | 59 | 60 | *A4t, area 4(trunk region)* | -13, -20, 73 | 15, -22, 71 |
| paracentral lobule | PCL_L(R)_2_1 | 65 | 66 | *A1/2/3ll, area1/2/3 (lower limb region)* | -8, -38, 58 | 10, -34, 54 |
|  | PCL_L(R)_2_2 | 67 | 68 | *A4ll, area 4, (lower limb region)* | -4, -23, 61 | 5, -21, 61 |
| supplementary motor area | SFG_L(R)_7_5 | 9 | 10 | *A6m, medial area 6* | -6, -5, 58 | 7, -4, 60 |
| postcentral gyrus (trunk) | PoG_L(R)_4_4 | 161 | 162 | *A1/2/3tru, area1/2/3(trunk region)* | -21, -35, 68 | 20, -33, 69 |
| postcentral gyrus ( upper limb, head and face region) | PoG_L(R)_4_1 | 155 | 156 | *A1/2/3ulhf, area 1/2/3(upper limb, head and face region)* | -50, -16, 43 | 50, -14, 44 |
|  | PoG_L(R)_4_3 | 159 | 160 | *A2, area 2* | -46, -30, 50 | 48, -24, 48 |
| premotor | SFG_L(R)_7_4 | 7 | 8 | *A6dl, dorsolateral area 6* | -18, -1, 65 | 20, 4, 64 |

**Supplementary Table 3.** The Demographic, Clinical Features and Detailed Information of Included Patients

| P/G | Age of seizure onset/ age at surgery | Etiology | Interictal EEG | Ictal EEG | Seizure type | Seizure semiology | MRI | MEG | PET | Baseline seizure(monthly) | Assumed seizure  foci | ****Past Surgical History**** |
| --- | --- | --- | --- | --- | --- | --- | --- | --- | --- | --- | --- | --- |
| 1/M | 7/14 | Encephalitis* | **Multiple sharp and slow waves in the left posterior frontal, central, and parietal regions.** | **Sharp and slow waves over the left posterior frontal and centroparietal regions. Muscle (EMG) artifact seen throughout all channels.** | FPC | **Right face and limb tonic-clonic** | **Left frontoparietal cortical atrophy** | Left central region | **Hypermetabolism in the left frontal and parietal lobes.** | 300 | Left frontal，parietal lobes | None |
| 2/M | 7/33 | MCD | **Generalized spike-wave and polyspike-wave discharges** | **Generalized low-voltage fast rhythm, spikes, and spike-and-slow-wave rhythm** | GES  GC | 1.Head orientation，bilateral limb tonic；  2. Head-and-neck epileptic spasms； | **Bilateral schizencephaly** | **Bilateral frontoparietal schizencephaly** | **Hypometabolism in the right frontal and left temporal lobes** | 75 | Bilateral frontal，parietal lobes | CC, VNS |
| 3/M | 1/19 | MCD | Frequent sharp and sharp-slow waves in frontal lobe and parietal lobe | **Low-voltage fast rhythm over the left frontal and parietal lobes** | FPC | 1.Right hand tonic-clonic；  2.eye blinking，bilateral–asymmetric  upper limbs tonic；  3. Head orientation  ,right upper limb clonic，right lower limb tonic；  4. eye blinking，right lower limb tonic； | **left precentral gyrus abnormal signal** | Left central region | Unavailable | 150 | **left precentral gyrus** | VNS |
| 4/F | 1/12 | MCD | Frequent sharp and sharp-slow waves **over the left parietal lobe, occipital lobe, and midline regions.** | Sharp waves over the left frontal lobe，temporal lobe and parietal lobe | FBTCS | 1.Right upper tonic，left upper limb tonic，bilateral tonic-clonic；  2.Confusion，left hand automatisms，head orientation，right upper tonic，bilateral tonic-clonic. | Left frontal lobe，parietal lobe，occipital lobe **schizencephaly** | Unavailable | **hypometabolism in the left frontal ,temporal, parietal, occipital lobes** | 10 | **Left central-parietal area.** | **Parietal lobe resection** |
| 5/M | 1/15 | unknown | Spike-and-slow-wave over left frontal and parietal | Slow wave over left frontal and temporal lobes | PIC, FBTCS | 1. Eye blinking，confusion；head retropulsion  2.Right upper limb tonic，bilateral tonic–clonic；  3. Head retropulsion，bilateral tonic- clonic. | **negative** | Bilateral parietal and frontal lobes | **Hypometabolism in left parietal and temporal**  **lobes** | 48 | **Bilateral frontocentral regions** | None |
| 6/M | 7/18 | MCD | Sharp waves over left frontal and parietal lobes | Sharp waves over left frontal and parietal lobes | PIC，FBTCS | 1.Right limb tonic-clonic；  2. Right limb tonic-clonic ，bilateral asymmetric  tonic-clonic seizure. | **Abnormal signal in the left central region** | Left central region | **Hypometabolism in left** parietal **and temporal lobes** | 4.5 | Left central region | None |
| 7/F | 2/15 | Encephalitis | **Generalized** spike-low waves | Spike-low waves over bilateral frontal lobes | FIC | 1.Confusion，bilateral–asymmetric upper limb tonic； | **Abnormal signal in the left frontal lobe** | Unavailable | **Hypometabolism in bilateral frontal, parietal lobes and left temporal lobe** | 300 | Left central region | None |
| 8/F | 2/20 | Progressive Myoclonus Epilepsies | **Bilateral spike-and-slow-wave** | **Bilateral synchronous 3-4 Hz spike-and-slow-wave** | GM | **Bilateral eyelid，facial and upper limb myoclonus** | negative | Bilateral frontal lobes，parietal lobe | **Hypometabolism in bilateral temporal lobes** | 150 | Bilateral frontal and parietal lobes | VNS |
| 9/F | 12/14 | Encephalitis | **Bilateral spike-and-slow-wave** | **Bilateral spike-and-slow-wave** | FPC | **Left face, forearm, and hand myoclonic** | Right frontal lobe，parietal lobe **schizencephaly** | Unavailable | **Hypometabolism in right frontal, parietal and temporal lobes** | 1500 | Right frontal and parietal lobes | None |
| 10/M | 8/27 | MCD | **Bilateral sphenoidal sharp waves** | **Fast wave rhythm over bilateral parietal lobe** | FPC，FBTCS | 1.Left **lower limb** somatosensory，tonic-clonic；  2. Left **lower limb** somatosensory，tonic-clonic，head orientation，eye deviation,  bilateral tonic-clonic seizure | **Bilateral medial parietal regions, adjacent to the paracentral lobule**  cortical atrophy and abnormal signal | Unavailable | **Hypometabolism in** bilateral parietal lobe, left temporal lobe, occipital lobe | 11 | Right central parietal region | None |
| 11/F | 8/13 | FCD Ib (**Previously pathologically confirmed)** | Bilateral frontal, parietal lobes spike-low wave | **Sharp and slow wave complexes with low-voltage fast activity over the right centroparietal region** | FIC, FBTCS | **1.left upper limb** myoclonic；  2.Bilateral upper limbs and left lower limb epileptic spasm；  3. Autonomic signs,eye deviation(left),left mouth corner tonic，left limb clonic；  4. **bilateral-asymmetric upper limbs** epileptic spasm；  5.Left upper limb epileptic spasm；  6.Left upper limb myoclonic, tonic, bilateral–asymmetric tonic-clonic. | Right frontocentral cortical dysplasia | Right frontal lobe, parietal lobe | **Hypometabolism in right frontal lobe** | 30 | Right central lesion | Right frontal lobe resection,  SEEG-RF |
| 12/M | 18/36 | MCD | Left frontal lobe slow waves；left temporal  Lobe sharp waves. | **low-voltage fast activity over the left temporal and** parietal | FBTCS | **right upper limb tonic，bilateral** tonic–clonic (figure-of-four) | **Left frontoparietal microgyria** | Left **temporal，frontal operculum，**parietal **operculum** | **Hypometabolism in left** parietal lobe ，left temporal，bilateral frontal lobe | 50 | Left frontal lobe, central region, parietal lobe | None |
| 13/M | 24/31 | MCD | Bilateral frontal lobe, left temporal, left **Sphenoidal slow waves** | SEEG showed independent left central region onset | FPC，FBTCS | **1.Right dorsal and lower limb** somatosensory, tonic-clonic，confusion.  2. **Right dorsal and lower limb** somatosensory, tonic-clonic, bilateral tonic-clonic seizure | **left frontal lobe cortical thickening** | Right **frontal operculum** | **Essentially symmetric metabolism in all cerebral lobes** | 15.5 | **Left paracentral lobule** | SEEG-RF |
| 14/M | 7/12 | MCD | Bilateral central region and parietal lobe spike wave and spike-slow wave | Bilateral central region and parietal lobe spike wave and spike-slow wave | FPC | Right lower limb somatosensory  (nonpainful)  , bilateral–asymmetric  four limbs atonic，hypersalivation | Negative | **Left paracentral lobe** | **Hypometabolism in left junction of frontal ，temporal，**parietal lobes。Bilateral **Superior parietal lobe** | 5 | **Left paracentral lobe** | None |
| 15/M | 8/23 | MCD | Bilateral spike- slow waves and sharp-slow waves | Low-amplitude fast activity over the left fronto-parietal region | FIC | 1.Right upper limb somatosensory,eye blinking  ,left hand automatisms  2. Head orientation  ,right limb tonic | **Gray matter heterotopia, suspected double cortex, abnormal signal in the right frontal lobe, and slightly increased signal in the left hippocampus.** | Bilateral frontal lobe and central region | **Hypermetabolism in the bilateral gray matter heterotopia.Hypometabolism in left temporal，bilateral junction of frontal and parietral lobes** | 2.5 | Left frontal lobe, temporal lobe, parietal lobe | SEEG-RC |
| 16/M | 6//23 | Unknown | Bilateral slow wave | **Intracranial electrodes revealed seizure onset characterized by low-amplitude fast activity in the left paracentral lobule.** | FPS | Dizziness, right limb and facial tonic-clonic. | Negative | Left insula lobe and **operculum of insula.** | **Hypometabolism in left paracentral lobe** | 175 | Left paracentral lobe | SEEG-RC |
| 17/F | 1/18 | MCD | Sharp waves over left temporal lobe，right central region，parietal lobe，temporal lobe | Independent ictal theta activity onset on bilateral frontal lobes | FIC | 1. Confusion，bilateral asymmetric  upper limb clonic；  2.Confusion,  oroalimentary automatisms  , gestural automatisms-distal. | **Bilateral perisylvian gray matter heterotopia** | Right frontal，temporal lobes | **Hypermetabolism in** left frontal lobe | 30 | **Bilateral perisylvian** | None |
| 18/F | 10/24 | HIE | Slow  waves and  sharp waves over right frontal lobe, central regions, temporal lobe | Spike and spike-slow waves over bilateral frontal  ,temporal, parietal lobes | GTC | Eye deviation  , bilateral–symmetric  tonic–clonic | **Bilateral temporal, parietal, and occipital lobe abnormal signals.** | Left temporal ,right frontal, temporal lobes | **Hypermetabolism in bilateral temporal lobe，frontoparietal junction** | 44 | Bilateral frontal lobe, temporal lobe and parietal lobe | None |
| 19/M | 5/16 | Traumatic brain injury | Shrap waves and sharp-slow waves over bilateral frontal and central region | Slow waves over right frontal lobe and central region | FBTCS,FIC | 1.Confusion，akinetic，gestural automatisms，bilateral–asymmetric  tonic–clonic;  2. Confusion | **Encephalomalacia in the right frontoparietal and temporal lobes, with surrounding gliosis** | Right insula lobe and central region | **Hypermetabolism in right frontal lobe and frontotemporal junction region** | 16 | Right frontal lobe and central region | **Right frontal glioma resection** |
| 20/M | 10/25 | MCD | Sharp waves over left parietal lobe | Spike and spike-slow waves over bilateral parietal lobes and central regions | FPC, FBTCS | 1.Right limb tonic-clonic;  2. Right limb tonic-clonic, head orientation,bilateral tonic-clonic. | Left central region cortical dysplasia | Left central region | **Hypermetabolism** in right tempral lobe and **hippocampus** | 0.5 | Right central region | None |
| 21/M | 2/29 | MCD | Spike waves and spike-slow waves over bilateral parietal lobe and occipital lobe | Spike waves and spike-slow waves over left frontal, parietal | FPC,  FBTCS | 1.Right lower limb somatosensory (nopainful)，tonic;  2. Right lower limb somatosensory, eye deviation, head orientation, bilateral tonic–clonic | Negative | Unavailable | **Hypermetabolism** in bilateral parietal and occipital lobes | 12.5 | Left parietal | None |
| 22/M | 18/32 | MCD | Sharp waves and slow waves over bilateral frontal lobe, central region and parietal lobe | Spike wave rhythms over the left posterior frontal, central, temporal and midline | FIC，FBTCS | 1.Right lower limb somatosensory(nopainful), tonic, eye deviation,confusion;  2.Right lower limb somatosensory(nopainful),tonic, eye deviation , bilateral–asymmetric tonic-clonic. | **Left frontal encephalomalacia with left parietal cortical atrophy** | Unavailable | **Hypermetabolism** in **left parietal lobe, temporal lobe, putamen, and thalamus.** | 80 | Left parietal | Left frontal lobe resection |

P: patient; G: gender; L: left; R: right; *Encephalitis with unknown etiology; F: female; M: male; FU: follow-up; FPC: Focal preserved consciousness seizure; FIC: Focal impaired consciousness seizure; FBTCS: Focal-to-bilateral tonic-clonic seizure; GC: Generalized clonic seizure; GTC: Generalized tonic-clonic seizure; GES: Generalized epileptic spasms; GM: Generalized myoclonic seizure; **HIE：Hypoxic-ischemic encephalopathy.CC: corpus callosotomy.**

**Supplementary Figure**


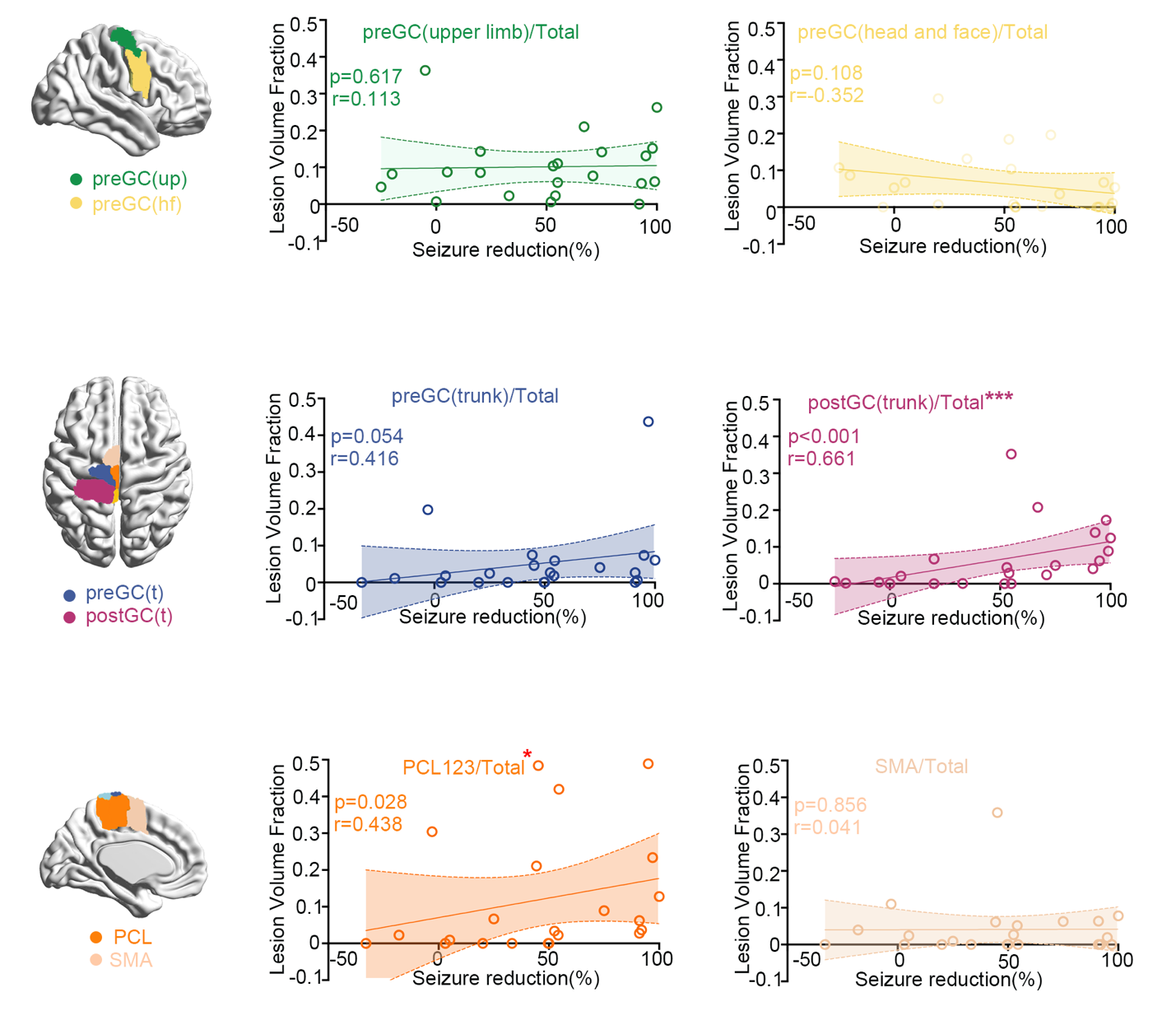


**Figure S1. Correlations between the proportion of total focus volume falling within each subregion and percent seizure reduction at 1 year.** *:P<0.05, ***:P<0.001.
